# Highlight: Unraveling the Origins of LUCA and LECA on the Tree of Life

**DOI:** 10.1093/gbe/evac072

**Published:** 2022-06-06

**Authors:** Casey McGrath

**Fig. 1.**
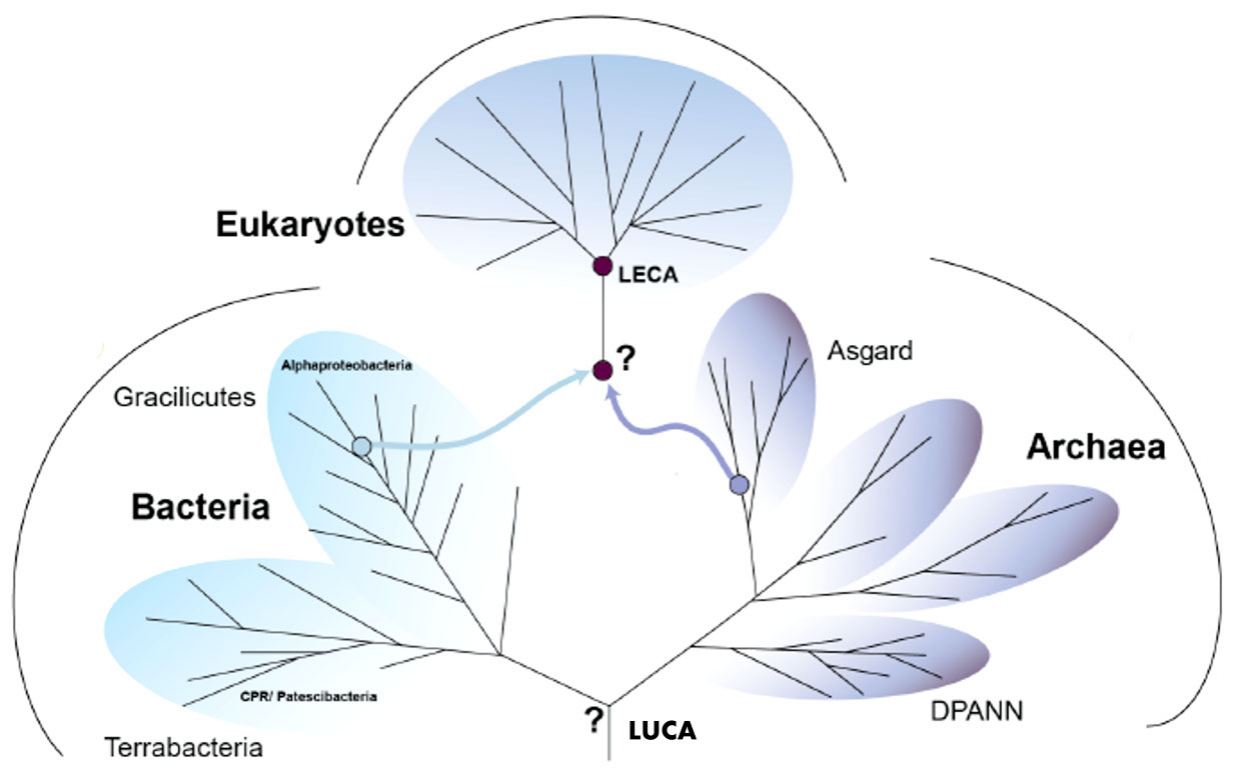
Tree of life. The tree of life contains three major branches—bacteria, archaea, and eukaryotes. Proposed locations for LUCA and LECA are shown. LUCA, last universal common ancestor; LECA, last eukaryotic common ancestor. Adapted from Spang et al. (2022).

The latest Virtual Issue from *Genome Biology and Evolution* highlights articles that provide new insight into the deep evolutionary relationships among extant organisms and the origin of eukaryotes from among archaeal lineages. All cellular organisms are descended from a shared ancestor, often referred to as LUCA—the last universal common ancestor. Relationships among these organisms can be depicted by an evolutionary network known as the “tree of life”, which for the past few decades has included three major forms of life—bacteria, archaea, and eukaryotes (fig. 1). Evolutionary biologists have long sought to understand the placement of LUCA within this framework, as well as the origin of LECA—the last eukaryotic common ancestor. Unfortunately, accurately inferring relationships among microbial lineages presents a major challenge due to the vast evolutionary distances involved, as well as the frequent lateral transfer of genetic material between lineages. Recently, however, new data and methods have resulted in profound changes to our understanding of the tree of life.

Headlining this Virtual Issue is a review from Anja Spang, Tara A. Mahendrarajah, Pierre Offre, and Courtney W. Stairs titled “Evolving perspective on the origin and diversification of cellular life and the virosphere” (Spang et al. 2022). Their article summarizes recent findings regarding LUCA and LECA, as well as the potential role of viruses in the evolution of both prokaryotes and eukaryotes. According to Spang, the authors are “fascinated by the concept of the tree of life as it has so much explanatory power, not only to describe the extant diversity of life and its relatedness but also to help understand genome evolution through time starting from LUCA. We therefore felt that a review integrating the various major discoveries regarding organismal and viral diversity as well as key evolutionary transitions would be very valuable.” In particular, the decision to include viruses in their discussion provides a somewhat unique perspective. According to co-author Mahendrarajah, “Viruses have rarely been discussed in reviews about the tree of life, and we felt they would be a great addition to our perspective considering their important roles in gene sharing between cellular life and the implications on organismal evolution.”

Several previously published investigations in this field focus on identifying key features that differentiate bacteria and archaea and attempting to infer their origins in the context of LUCA. For example, bacteria and archaea possess different cell membrane phospholipids that are synthesized by non-homologous enzymes, and Coleman et al. (2019) showed that LUCA likely possessed the ability to synthesize archaeal-type membrane phospholipids. Bacteria and archaea also differ in their histone proteins, although relatively little remains known about archaeal histone proteins. Stevens et al. (2022) demonstrated that the two histones present in the model archaeon *Thermococcus kodakarensis* are conserved at least across the order Thermococcales and also revealed the presence of highly diverged histone-fold proteins related to bacterial histones in several Thermococcales genomes. The origin of nitrogenases, enzymes that fix nitrogen and are found in both bacteria and archaea, has also puzzled many scientists; a recent study by Garcia et al. (2022) showed that nitrogenases may have evolved from maturases, homologs that today participate in nitrogenase cofactor assembly, rather than the other way around. This raises new questions about the environmental factors that led to the origin of this critical biogeochemical innovation.

Another primary focus in the field is the position of LUCA relative to extant bacteria and archaea. While it is generally assumed that the root of the tree of life lies in between archaea and bacteria, it is difficult to formally rule out alternatives due to the potential for phylogenetic artifacts. Furthermore, the root of the archaea and in particular the placement of the diverse DPANN archaea (an acronym based on the first five groups discovered: Diapherotrites, Parvarchaeota, Aenigmarchaeota, Nanoarchaeota, and Nanohaloarchaeota), which represent many lineages with symbiotic and small genome members, remains uncertain (Spang et al. 2022). It is particularly challenging to accurately place symbionts in phylogenetic trees among others because they can exchange genes with their hosts and often experience faster evolutionary rates, which in turn can confound tree-building methods. For example, Feng et al. (2021) showed that some individual gene trees grouped one of the DPANN lineages, the Nanohaloarchaea, with the Haloarchaea, rather than with other DPANN taxa. Despite this, they found that most large concatenated data sets were consistent with the monophyly of the DPANN superphylum in unrooted phylogenies, highlighting the difficulty in correctly inferring deep relationships among these lineages. While various concatenated gene tree inferences suggest that the DPANN form a monophyletic clade, it remains to be assessed whether they are monophyletic and deep-branching in rooted trees (Spang et al. 2022).

A further key question pertains to the origin of eukaryotes and the placement of LECA on the tree. A symbiogenetic origin of eukaryotes has long been suspected, in which an archaeal cell acquired a bacterial endosymbiont, resulting in a proto-eukaryote containing a proto-mitochondrion. According to Tria et al. (2021), evidence from gene duplications in LECA support this early origin of mitochondria, revealing serial copying of bacterial genes from the proto-mitochondrion to the archaeal host’s genome. This may partially explain why a study by Brueckner and Martin (2020) found that eukaryotic genomes generally possess more bacterial than archaeal genes, with bacteria contributing 53% of the genes in eukaryotic lineages without plastids and 61% in photosynthetic eukaryotic lineages.

As detailed in the Spang et al. (2022) review, phylogenetic analyses have recently suggested that the closest archaeal sister lineage to eukaryotes may be the Asgard archaea, a proposed superphylum consisting of the Lokiarchaeota, Thorarchaeota, Odinarchaeota, and Heimdallarchaeota. As further evidence for this, Penev et al. (2020) discovered that the large ribosomal subunit (LSU) rRNA of the Lokiarchaeota and Heimdallarchaeota bridge the gap in size between prokaryotic and eukaryotic LSU rRNAs. This phylogenetic grouping remains somewhat controversial however due to the fact that the vast majority of Asgard sequences come from metagenome-assembled genomes (MAGs), which can suffer from data analysis artifacts that may result in spurious findings. Indeed, a study by Garg et al. (2021) suggested that some Asgard archaeal MAGs could be unnatural constructs resulting from the assembly or binning processes. Nevertheless, this remains debated (Spang et al. 2022), and the first genome of a cultivated member of the Lokiarchaeota (Imachi et al. 2020) has confirmed the ubiquitous presence of eukaryotic signature proteins in Asgard archaea.

Additional research has focused on early eukaryotic evolution and the features that date back to LECA, with many studies showing that LECA already possessed a relatively high degree of eukaryotic complexity. This includes several proteins and protein complexes involved in cellular processes thought to be unique to eukaryotes. For example, Vargová et al. (2021) revealed that LECA possessed as many as 16 ARF GTPases, proteins that play a role in eukaryote-specific processes such as membrane trafficking, tubulin assembly, actin dynamics, and cilia-related functions. Similarly, Yoshinaga and Inagaki (2021) found that LECA likely possessed two distinct SMC proteins, which are critical for successful chromosome replication and segregation, whereas Santana-Molina et al. (2021) showed that LECA already possessed a CATCHR protein complex involved in vesicle trafficking. One critical question is whether such features may have originated in the ancestral archaeal lineage prior to eukaryogenesis. Knopp et al. (2021) found that only 0.3% of protein families present in LECA can be attributed to the Asgard archaea, casting doubt on the idea that the ancestral archaeal lineage evolved many of these features prior to eukaryogenesis. Investigating another aspect of LECA, Skejo et al. (2021) intriguingly suggested that LECA may have been multinucleate, pointing to the commonality of multinucleated forms across eukaryotic supergroups. If true, this would fundamentally alter our understanding of the evolutionary transitions among early eukaryotes.

Spang et al. (2022) suggest a number of ways to further advance our understanding of the tree of life and resolve the unanswered questions that remain. According to co-author Stairs, progress thus far “has been made possible by major technological leaps in sequencing and bioinformatics pioneered by the microbiological and medical fields. Our next challenge is to improve methods for eukaryotic genome assembly, gene prediction, and gene annotation as eukaryotic genomes are often more complex with higher levels of sequence divergence than their prokaryotic counterparts.” Other critical areas of exploration include increasing the amount of sequence data available across the tree of life, developing new phylogenetic and phylogenomic methods to resolve incongruencies and uncertainties, reconstructing ancestral sequences and genomes, and using cell biology to link genotypes to phenotypes and better understand protein structures and cellular features. Co-author Offre believes that such methodological approaches are necessary for “further progress in our understanding of the tree of life in the years to come,” ultimately leading to “an integrative view on life’s biodiversity and its evolution.”
